# Hepato-Intestinal Toxæmia.—I

**Published:** 1909-05-22

**Authors:** 


### SPECIAL ARTICLE.

### HEPATO-INTESTINAL TOXÆMIA.—I.

Whether or not auto-intoxication by the absorption of poisonous products from the alimentary canal really plays so important a part in the production of symptoms as some observers would have us believe, is still a matter of conjecture and controversy. Notwithstanding bacteriological investigation of fæces, chemical analysis of urine for such substances as the conjugated sulphates, indican, putrescine, and so forth, it is still quite an open question whether there is any such thing as an intestinal auto-intoxication in the generally accepted sense at all. There are numerous clinical symptoms that are described as those of hepato-intestinal-toxæmia by those who are staunch upholders of the intestinal auto-intoxication theory. There is little or no doubt about the symptoms; the moot point is whether they are due to the cause to which they are attributed.

Dr. Edward Quintard, Professor of Medicine in the New York Post-Graduate Medical School and Hospital, has written upon the subject at some length, and mainly from the clinical and the therapeutical points of view. He states that he has seen cases at all ages and in both sexes, but that the large proportion are between 30 and 50 years of age. Men seem to suffer more frequently than women. Cases are both acute and chronic, but the majority belong to the latter category, lasting many weeks or months, and not infrequently extending into years.

Their general appearance may be very fair, their symptoms being mainly subjective, at first at any rate, and in those who suffer from the complaint in slighter degree. There is often a mild grade of anæmia, or a sallowness without actual anæmia. The conjunctivæ are not jaundiced, but they are decidedly not white; perhaps the term icteroidal might describe them. The tongue is coated yellowish-white or yellowish-grey; this coating may be thin or thick, extending over the entire tongue or limited to the back of it. There is often some dryness of the mouth also. The patients may be fairly well nourished, but in the chronic cases, where the supposed toxæmia has seriously affected the general metabolism and nervous system, marked emaciation may exist. They not infrequently present a rather listless, depressed, or melancholic attitude, although this is not constant. On questioning them, it will be found that, except in very severe or unusual cases, symptoms begin in a characteristic way and run a definite course until ultimately the nervous system suffers, and to the symptoms caused by the chronic poison must be added those of neurasthenia.

The patients state that their condition is one of constant ill-health, with certain periods when they feel particularly bad; or else they feel in good health for a few weeks or possibly months, and then suffer attacks which render them out of sorts for a longer or shorter period. In the intermittent cases the onset of the attack may be preceded by a 24 hours' period during which the patient feels more than ordinarily buoyant and energetic, with an increase in appetite and a desire to be up and doing—a condition of particular *bien être*. The following symptoms then manifest themselves, at times one and again another being in primary evidence.

An indescribable discomfort in the right upper abdominal quadrant. It is not a pain, nor is there any particular spot of sensitiveness to pressure in this region; yet the patients frequently complain that they feel as if they wished to "get away from something" in this neighbourhood, and at times they will exert deep manual pressure over this area in order to see whether by so doing this peculiar discomfort can be relieved. Whether or not a mild duodenitis is at the root of this cannot be definitely said, but at any rate the symptom is a fairly constant one, and it may precede all other symptoms by 24 hours or more. Like the other symptoms, it is not invariably present.

Circulatory and respiratory disturbances may be absent, slight, or severe. Dizzy sensations, or feelings of "light-headedness," are not uncommon. This dizziness may be limited to times when the patient makes a sudden movement, such as rising from bed or from a sitting posture, or when he suddenly turns his head. At times, however, dizziness and vertigo may be so severe as to prevent the patient from raising his head at all, or they may temporarily interfere with his gait. He may bump into doors and walls whilst walking, and this may greatly alarm both his friends and himself.

Palpitation may come on at any time, especially after meals or on exertion. The attacks may be very severe and alarming, being associated sometimes with sudden dyspnœa and feeling of impending dissolution. So sudden and unexpected may these attacks sometimes be that the medical practitioner himself, has he not seen such cases before, may believe that some obscure cardiac lesion exists, until he has convinced himself of the contrary by most thorough and repeated examinations.

In these attacks of dyspnœa the patient feels that he cannot take a deep breath; his heart may palpitate; arrhythmia may occur; the patient may wake up with such an attack, or he may be walking in the street when seized. Angina pectoris may be simulated. The patient's first sensation may be that of a warm wave or flush passing suddenly over his entire body. Immediately upon this he will be seized with dyspnœa, palpitation, oppression in the chest, and feeling of imminent death. In some, dyspnœic attacks may occur but once, or again they may recur more or less frequently for a time and last for from a few minutes to an hour or longer. At times breathlessness on exertion is another marked symptom of both the acute and chronic form of the condition. Oddly enough, in cases in which it is manifest it becomes especially marked after a dose of a mercurial, particularly after calomel. It would seem that in such cases the sudden unloading of the poison from the liver and its re-absorption from the intestinal tract into the circulation increase the toxæmia for the time being, and so affect the neuromuscular system that any effort or exertion disturbs the respiratory and circulatory equilibrium. Such attacks are more apt to occur in neurasthenic patients.

These are varied, and mild or severe according to the degree of toxæmia and the susceptibility of the nervous organism of the patient. Headache of the migraine type occurs, but less commonly than do feelings of congestion, fulness or lightness in the head, or a dull heavy ache in the occipital region. Apart from the temporary increased sense of well-being described above as a prodromal sign of an attack, the patients are generally despondent and depressed. When such a chronic toxæmia is superimposed upon a system that is already neurasthenic or exhausted there may be great difficulty in persuading either the patient or his friends to take a hopeful view of the case. The marked depression and despondency cause them to believe that there is something the matter with their minds, and they either dread that they will become insane or believe that they have actually become so.

For months at a time these patients may feel giddy or uncertain when going downstairs. They feel as if they might pitch forward and fall. They may also complain of other subjective sensations, such for instance as a feeling as if the pavement were suddenly rising up towards them as they walk. Doubtless the toxæmia is causing errors in the cerebral circulation in these cases; sometimes it is really difficult to decide whether there may not be a more serious labyrinthine or other intracranial lesion. Insomnia and bad dreams, especially the sensation of sudden falling just as sleep approaches, are common symptoms also.

So far as the stomach itself is concerned, in the vast majority of cases there are no subjective or objective signs, unless perhaps impairment of appetite. Neither the physical nor the functional signs reveal anything abnormal in the stomach as a rule. It is different, however, with the liver and with the intestinal tract. One looks for and usually finds evidence of constipation, various fermentations, and intestinal putrefaction; the stools are light and almost clay-like, though there is no jaundice and the urine contains no bile pigments; fat is poorly absorbed; and there is decided discomfort in the right upper quadrant of the abdomen.

We have, then, in the intermittent type of the disease periods of seemingly perfect health interrupted by attacks that usually have a characteristic onset, and last for from several days to as many weeks. Occasionally, however, the condition is one which, instead of ending at this time, runs a subacute course for a month or so; or at any time the intermittent may change to the remittent type.

The attacks in the intermittent type begin suddenly. The patient may have a period of hypereuphoria during which for a few hours or a whole day he feels particularly buoyant and energetic; then he notices that his stools are much lighter in colour than they were, that he has a discomfort in the right upper quadrant of the abdomen; that his tongue becomes coated, his face sallow, and his conjunctivæ icteroidal, the last three especially in the morning. At the outset he may suffer from polyuria, and the urine may be very light in colour or be voided in much less quantity than usual. He feels decidedly depressed and lacking in physical and mental energy; he may or may not feel drowsy; he may or may not have nausea; he very rarely has vomiting; his feet and hands become cold, he has chilly sensations, and it may be impossible for him to become properly warm all over; he has sensations of dizziness or of vertigo. He may have several of these symptoms or all of them. There may be numb sensations in different parts of the body, especially in the legs and forearms. At times there is difficulty in enunciating words—not a true aphasia, but a condition in which the patient feels as though his tongue were swollen and thick, so that he pronounces words imperfectly, especially if he speaks in a hurry. During such times, also, his memory is not keen; he cannot concentrate his thoughts, and he may find it very difficult to attend to his affairs. (*To be concluded.*)

